# Microbial Biotransformation of Plant Bioactive Compounds: Mechanisms, Enhanced Bioactivities, and Applications in Functional Food Development

**DOI:** 10.3390/foods15213894

**Published:** 2026-10-30

**Authors:** Richan Fang, Futao Liu, Yajuan Chen, Junjie Li, Jiating Lin, Youlin Cheng, Jianmin Tang, Jun Guo, Xiangxin Li, Weiwei Li

**Affiliations:** 1 Faculty of Life Science and Technology, Kunming University of Science and Technology, Kunming 650500, China; 18778778115@163.com (R.F.); liufutao@stu.kust.edu.cn (F.L.); cyj3347982126@163.com (Y.C.); lijunjie0307@126.com (J.L.); guojun0591@126.com (J.G.); 18269405339@163.com (J.L.); cyl43360@163.com (Y.C.); 18313548621@163.com (J.T.); 2 State Key Laboratory of Resource Insects, Institute of Apicultural Research, Chinese Academy of Agricultural Sciences, Beijing 100093, China; lixiangxin@caas.cn; 3 Yunnan Key Laboratory of Biodiversity Information, Kunming Institute of Zoology, The Chinese Academy of Sciences, Kunming 650201, China

**Keywords:** plant-derived bioactive compounds, microbial biotransformation, fermentation, microbial enzymes, functional foods, multi-omics

## Abstract

Plant-derived bioactive compounds, including polyphenols, flavonoids, alkaloids, terpenoids, and saponins, possess diverse health-promoting properties but are often limited by poor bioaccessibility, structural complexity, and low stability. Microbial biotransformation provides a green and food-compatible strategy for modifying these compounds through enzymatic reactions and metabolic processes, generating derivatives with improved accessibility and potential functional properties. This review summarizes recent advances in microbial transformation mediated by lactic acid bacteria, *Bacillus*, yeasts, and filamentous fungi, focusing on key transformation pathways, including deglycosylation, hydrolysis, oxidation–reduction, methylation, and glycosylation, as well as their enzymatic mechanisms. The applications of transformed products in fermented foods, functional ingredients, and nutraceuticals are discussed, along with their potential antioxidant, anti-inflammatory, metabolic regulatory, and bioavailability-related effects. Current challenges, including incomplete mechanistic understanding, process standardization, safety evaluation, scale-up, and regulatory issues, are highlighted. Future integration of multi-omics, synthetic biology, enzyme engineering, and intelligent fermentation technologies will enhance the efficiency and industrial potential of microbial transformation systems for developing safe and evidence-based functional food ingredients.

## 1. Introduction

Plants are important sources of bioactive compounds that are widely used in foods, medicinal products, and dietary supplements. Plant secondary metabolites, including phenolic compounds, flavonoids, alkaloids, terpenoids, and saponins, exhibit diverse biological activities related to oxidative stress regulation, inflammation modulation, and metabolic homeostasis [1,2,3,4]. Among plant bioactive compounds, polyphenols and flavonoids are among the most extensively investigated because of their diverse biological activities, including antioxidant effects and modulation of multiple cellular and molecular pathways [5,6,7]. However, the functional application of many phytochemicals remains restricted by their intrinsic physicochemical and pharmacokinetic limitations [8,9]. Flavonoids predominantly occur in plants as glycosylated derivatives, and their intestinal absorption and bioavailability are strongly influenced by glycosylation status, molecular size, lipophilicity, solubility, and interactions with intestinal transporters and metabolic enzymes [9,10]. Similarly, many phenolic acids and tannins exhibit considerable biological activities in vitro, but their bioavailability in vivo can be limited by structural complexity, low solubility, extensive phase I and phase II metabolism, and transformation by the gut microbiota [11,12]. Structural modification has therefore been explored to improve the utilization of plant bioactive compounds. Approaches including extraction optimization, encapsulation, enzymatic modification, and chemical derivatization have been investigated [8,13,14]. However, chemical modification may involve relatively harsh reaction conditions and limited regioselectivity, whereas isolated enzymatic approaches can be constrained by enzyme cost, stability, catalytic efficiency, and substrate compatibility [14,15].

Microbial biotransformation provides an alternative strategy by using whole microbial cells or microbial-derived enzymes to catalyze selective structural modifications of plant bioactive compounds under relatively mild and potentially food-compatible conditions [16,17]. In this context, microbial biotransformation is precisely defined as the enzyme- or metabolism-driven conversion of plant-derived compounds into structurally modified derivatives through specific catalytic reactions, resulting in chemically distinct products with altered physicochemical or biological properties. This process should be clearly distinguished from conventional fermentation and from simple extraction or matrix release. Fermentation primarily refers to the overall microbial growth and metabolic process used for food preservation, flavor development, or nutritional modification. While biotransformation may occur as one of the biochemical events during fermentation, fermentation itself does not necessarily involve targeted structural modification of specific phytochemicals. In contrast, simple extraction or matrix disruption merely increases the release or recovery of compounds already present in plant materials, without altering their chemical structures. Microbial biotransformation, however, generates new molecular entities through enzyme-catalyzed reactions, including deglycosylation, ester hydrolysis, decarboxylation, oxidation–reduction, and glycosylation, thereby producing structurally modified metabolites [18]. These enzyme-mediated reactions enable the selective modification of complex phytochemicals under relatively mild reaction conditions. A broad range of microorganisms, including lactic acid bacteria (LAB), Bacillus species, yeasts, and filamentous fungi, have been investigated for the biotransformation of plant-derived compounds [16,19,20]. LAB are particularly attractive for food and functional-food applications because of their extensive history of use in fermented foods and their diverse enzymatic capacities [20,21].

In recent years, microbial-transformation-driven fermentation has attracted increasing attention in functional food and nutraceutical development. Fermentation of plant-based beverages, cereals, and other plant matrices can substantially alter metabolite profiles and, depending on the microbial strain and processing conditions, improve the bioaccessibility, bioavailability, and functional properties of selected bioactive compounds [22,23,24]. Microbial conversion of phenolics during fermentation can generate structurally distinct metabolites, including aglycones, phenolic acid derivatives, and other low-molecular-weight products, thereby expanding the diversity of potentially functional metabolites [25]. Microbial transformation may also generate novel metabolites through secondary metabolism, providing opportunities for the development of value-added functional ingredients [26].

Despite substantial progress, challenges remain in translating microbial biotransformation from laboratory studies to industrial applications. These challenges include incomplete elucidation of strain-specific transformation pathways, substantial strain- and substrate-dependent variability, safety and regulatory assessment, and difficulties associated with process standardization, productivity, downstream processing, and scale-up [26,27,28,29]. Future studies should therefore incorporate quantitative parameters, including substrate conversion rate, product yield and titer, metabolite concentration, reaction time, volumetric productivity, and fermentation scale, to facilitate objective comparison among transformation systems and to provide more meaningful assessments of their technological and industrial feasibility.

Therefore, as illustrated in Figure 1, this review summarizes recent advances in microbial biotransformation of plant-derived bioactive compounds, focusing on microbial resources, enzymatic mechanisms, functional consequences, and applications in functional food development. The roles of bacteria, yeasts, and filamentous fungi are compared, followed by discussion of major transformation pathways and their effects on the physicochemical and biological properties of plant metabolites. Emerging strategies involving multi-omics, metabolic engineering, synthetic microbial communities, and artificial intelligence are also discussed, with emphasis on improving transformation efficiency, reproducibility, safety, and industrial applicability [22,30].

## 2. Plant Bioactive Compounds as Substrates for Microbial Biotransformation: Structural Diversity and Functional Potential

Plants produce a wide range of chemically diverse secondary metabolites that represent valuable resources for functional food development. These compounds are synthesized through complex metabolic pathways and participate in plant defense mechanisms, environmental adaptation, and ecological interactions [31]. Table 1 summarizes the classification and functional potential of plant-derived bioactive compounds as substrates for microbial biotransformation.

### 2.1. Polyphenols as Major Substrates for Microbial Transformation

Polyphenols are characterized by aromatic rings containing multiple hydroxyl groups and are widely distributed in fruits, vegetables, cereals, tea, coffee, herbs, and bee-derived plant materials [11]. According to their chemical structures, polyphenols can be categorized into phenolic acids, flavonoids, stilbenes, lignans, and tannins [32]. The biological functions of polyphenols are mainly associated with their electron-donating capacity, metal-chelating ability, and regulation of oxidative-stress-related signaling pathways [33].

During fermentation, microorganisms produce various functional enzymes, including esterases, tannases, decarboxylases, and oxidoreductases, which participate in phenolic degradation and structural modification [34]. Tannase-producing microorganisms hydrolyze ester bonds in hydrolysable tannins, releasing gallic acid and other low-molecular-weight phenolic compounds with improved biological accessibility [35]. Phenolic acid decarboxylases further convert hydroxycinnamic acids into vinyl derivatives, contributing to altered antioxidant and antimicrobial properties. In fermentation systems containing phenolic-rich substrates, conversion efficiency is strongly influenced by strain specificity, substrate concentration, pH, and fermentation time.

### 2.2. Flavonoids and Isoflavonoids: Structural Remodeling by Microbial Enzymes

Flavonoids constitute the largest subclass of plant polyphenols and include flavonols, flavones, flavanones, flavan-3-ols, anthocyanins, and isoflavonoids. Their conserved C6-C3-C6 skeleton provides multiple modification sites, making them highly suitable substrates for microbial enzymatic transformation. Microbial deglycosylation is therefore considered a key transformation pathway. Specific β-glucosidases from microorganisms such as *Lactiplantibacillus plantarum* and *Lacticaseibacillus rhamnosus* catalyze the hydrolysis of flavonoid glycosidic bonds, while fungal microorganisms such as *Aspergillus niger* have also been extensively investigated for flavonoid glycoside transformation [36,37]. Several studies have reported increased antioxidant capacity or altered biological activities of the resulting aglycones compared with their glycosylated precursors; however, the extent and direction of these changes depend on the specific compound, assay system, microbial strain, and transformation conditions.

Beyond hydrolysis, microorganisms can catalyze diverse structural modifications, including hydroxylation, methylation, glycosylation, hydrogenation, and C-ring cleavage. Filamentous fungi, particularly *Cunninghamella*, *Aspergillus*, and *Penicillium*, have been extensively investigated for these flavonoid biotransformation reactions, including hydroxylation, O-methylation/O-demethylation, hydrogenation, glycosylation, and C-ring cleavage [38]. Because some species and strains of *Aspergillus* and *Penicillium* can produce mycotoxins, their application in functional-food systems requires careful strain-level identification, characterization of secondary-metabolite profiles, and verification of the absence of relevant toxigenic potential [39].

### 2.3. Phenolic Acids and Their Microbial Conversion Pathways

Phenolic acids, including hydroxybenzoic acids and hydroxycinnamic acids, are widely distributed in plant-based foods. Representative compounds include gallic acid, caffeic acid, ferulic acid, and p-coumaric acid. Microbial transformation can modify functional groups of phenolic acids through decarboxylation, reduction, hydroxylation, and oxidation reactions. Phenolic acid decarboxylases produced by microorganisms convert hydroxycinnamic acids into vinyl derivatives, whereas oxidoreductases participate in structural modifications that alter their biological properties [40].

Specific microbial strains have been identified for these reactions. For example, *Lactiplantibacillus plantarum* and *Levilactobacillus brevis* possess phenolic acid decarboxylases that convert hydroxycinnamic acids, including ferulic acid and *p*-coumaric acid, into their corresponding vinyl derivatives [23]. Esterase- and decarboxylase-producing microorganisms, including selected strains of *Bacillus subtilis*, can also transform or release phenolic acids from plant-derived substrates [41]. The efficiency of these transformations depends on substrate availability, enzyme activity and specificity, microbial strain characteristics, and fermentation conditions.

Fermented foods, including sourdough, tea beverages, soybean products, and plant-based beverages, contain abundant microbial-derived phenolic metabolites. Increasing evidence indicates that the functional properties of fermented plant foods are often associated with transformation products rather than original plant compounds. Therefore, identification of key enzymes and transformation pathways has become an important research direction for functional food development.

### 2.4. Alkaloids as Specialized Substrates for Microbial Biotransformation

Alkaloids are nitrogen-containing specialized metabolites widely distributed in medicinal plants, edible plants, beverages, and spices. Representative food-related alkaloids include caffeine, theobromine, capsaicin-derived compounds, and berberine. Compared with polyphenols, alkaloids possess more complex heterocyclic nitrogen-containing structures, which contribute to their diverse biological activities but also create challenges for their application in functional food systems. Therefore, microbial biotransformation provides an attractive strategy for selectively modifying alkaloid structures and improving their physicochemical and functional properties [42].

Microorganisms can catalyze various structural modifications of alkaloids, including hydroxylation, demethylation, oxidation, reduction, and glycosylation reactions. For caffeine transformation, specific microorganisms, particularly *Pseudomonas putida* and other *Pseudomonas* species, have been reported to produce caffeine-demethylating enzymes, including the N-demethylases NdmA, NdmB, and NdmCDE, which sequentially remove methyl groups from caffeine and generate theobromine, paraxanthine, 7-methylxanthine, and ultimately xanthine [43,44,45]. In contrast, filamentous fungi, including *Aspergillus* species, generally follow a different N-demethylation route in which caffeine is initially converted to theophylline and subsequently to 3-methylxanthine and other downstream metabolites [46]. These transformations have been demonstrated primarily in laboratory-scale microbial cultures and enzyme or whole-cell systems. Further optimization of enzyme activity, cofactor supply, process stability, reaction productivity, and product recovery is therefore required for efficient biotechnological application. Although microbial transformation of alkaloids remains less extensively investigated than that of polyphenols and flavonoids, recent advances in genome mining, metagenomics, and enzyme engineering have provided new opportunities for discovering alkaloid-transforming microorganisms and developing functional alkaloid derivatives for food and nutraceutical applications.

### 2.5. Terpenoids and Saponins as Emerging Transformation Substrates

Terpenoids and saponins represent important groups of plant-derived functional compounds with considerable potential for functional food development. Terpenoids comprise structurally diverse metabolites, including monoterpenes, diterpenes, triterpenes, and carotenoids, whereas saponins are mainly composed of glycosylated triterpenoid or steroid structures commonly found in medicinal plants, legumes, and edible botanical resources.

Microbial transformation provides an effective approach for improving the functional characteristics of terpenoids and saponins through selective enzymatic modifications, including glycosylation, deglycosylation, hydroxylation, and oxidation. Strains including *Lactiplantibacillus plantarum*, *Aspergillus niger*, and *Aspergillus tubingensis* have been reported to produce β-glucosidases capable of hydrolyzing sugar residues from major ginsenosides. Ginsenosides from Panax ginseng are among the most extensively studied examples of microbial saponin transformation. Specific microorganisms and their glycosidases can sequentially hydrolyze sugar residues from major ginsenosides, such as Rb1, Rb2, and Rc, resulting in the formation of minor ginsenosides, including compound K [47]. For example, microbial conversion of ginsenoside Rb1 to compound K has been achieved with different microorganisms, with reported conversion efficiencies depending strongly on strain selection, substrate concentration, enzyme activity, and reaction time [48].

### 2.6. Other Plant-Derived Bioactive Compounds as Transformation Targets

Beyond the major classes of plant bioactive compounds discussed above, various other specialized metabolites, including glucosinolates, lignans, pigments, and polysaccharides, also represent valuable substrates for microbial transformation. Their structural diversity provides additional opportunities for microbial enzymes to generate novel derivatives with improved physicochemical properties and functional characteristics.

Glucosinolates, which are abundant in cruciferous vegetables, can be transformed by microbial enzymes into isothiocyanate derivatives through hydrolysis reactions. These microbial-derived metabolites contribute to the characteristic flavor formation of cruciferous foods and possess important functional potential. Intestinal and food-associated microorganisms containing myrosinase-like activity can promote glucosinolate hydrolysis. However, the application of microbial glucosinolate transformation in functional foods remains dependent on strain safety evaluation and controlled fermentation processes [49]. Similarly, plant pigments, including anthocyanins and betalains, can undergo microbial degradation and structural remodeling, producing smaller phenolic metabolites with altered stability, solubility, and biological properties [50,51]. Polysaccharides represent another important category of transformation substrates because their structural complexity determines their physicochemical and functional characteristics. During microbial fermentation, extracellular enzymes, such as glycosidases, cellulases, and hemicellulases, can modify polysaccharide molecular weight distribution, branching patterns, and functional groups. These structural alterations may influence their fermentability, interaction with gut microorganisms, and potential applications as functional food ingredients [52].

**Table 1:** Classification and functional potential of plant-derived bioactive compounds as substrates for microbial biotransformation.

Plant Bioactive Compound class	Representative Compounds	Representative Microbial Strains	Key Enzymes Involved	Major Transformation Reactions/Products	Functional Improvements After Transformation	Representative References
Polyphenols	Caffeic acid, ferulic acid, chlorogenic acid, tannins	*Lactiplantibacillus plantarum*, *Lacticaseibacillus rhamnosus*, *Aspergillus niger*, *Bacillus subtilis*	Esterase, tannase, phenolic acid decarboxylase	Ester hydrolysis of phenolic esters; tannin degradation releasing gallic acid; decarboxylation of hydroxycinnamic acids producing vinyl derivatives	Increased release of free phenolic compounds; altered antioxidant properties; improved accessibility of phenolic metabolites	[34,35]
Flavonoids	Quercetin, rutin, catechin, anthocyanins	*Lactiplantibacillus plantarum*, *Lacticaseibacillus rhamnosus*, *Aspergillus oryzae*, *Aspergillus niger*	β-Glucosidase, α-rhamnosidase, glycosyltransferase	Deglycosylation of flavonoid glycosides; conversion of rutin to quercetin derivatives; formation of flavonoid aglycones	Generation of aglycones with altered solubility and absorption characteristics; biological effects require experimental validation	[36,37,38]
Isoflavonoids	Daidzin, daidzein, genistin, genistein	*Lactiplantibacillus plantarum*, *Bifidobacterium longum*, *Lactobacillus casei* reduces biological accessibility	β-Glucosidase, β-galactosidase	Hydrolysis of isoflavone glycosides to aglycones such as daidzein and genistein	Increased formation of absorbable isoflavone aglycones; enhanced estrogenic and metabolic regulatory potential	[36,37]
Alkaloids	Caffeine, berberine, capsaicin-related compounds	*Pseudomonas putida*, *Aspergillus* spp., engineered microbial strains	Demethylase, oxidoreductase, hydroxylase	Demethylation, oxidation, hydroxylation, and structural modification of alkaloids	Modified physicochemical properties; potential reduction in undesirable sensory effects; improved functional utilization	[23,40,41]
Terpenoids and saponins	Ginsenosides (Rb1, Rd), triterpenoids, carotenoids	*Aspergillus niger*, *Aspergillus oryzae*, *Bacillus subtilis*, *Lactobacillus* spp	β-Glucosidase, glycosidase, glycosyltransferase, oxidoreductase	Sequential deglycosylation of ginsenosides; conversion of major ginsenosides into minor metabolites such as compound K	Improved absorption characteristics and generation of highly active secondary metabolites	[42,43,44,45,46]
Other plant-derived compounds	Glucosinolates, anthocyanins, betalains, polysaccharides	*Lactiplantibacillus plantarum*, *Bacillus subtilis*, filamentous fungi	Myrosinase, cellulase, hemicellulase, glycosidase	Hydrolysis of glucosinolates; pigment degradation; polysaccharide depolymerization and structural remodeling	Modified antioxidant capacity; altered gut microbiota interactions; enhanced functional food applications	[49,50,51,52]

## 3. Microbial Strategies and Transformation Mechanisms of Plant Bioactive Compounds

### 3.1. Lactic Acid Bacteria-Mediated Transformation of Plant Bioactive Compounds

Lactic acid bacteria (LAB), including *Lactobacillus*, *Lactiplantibacillus*, *Levilactobacillus*, *Pediococcus*, and *Lactococcus*, are widely used in plant-based fermentation because of their established food applications, safety profiles, probiotic potential, and metabolic adaptability [23,37]. Beyond acidification and preservation, LAB can selectively transform plant phytochemicals through enzymatic reactions under relatively mild conditions, converting conjugated compounds into structurally modified derivatives. LAB-mediated transformation mainly involves β-glucosidases, β-galactosidases, esterases, and phenolic acid decarboxylases, which catalyze the cleavage or modification of glycosidic and ester bonds. Among these pathways, β-glucosidase-mediated deglycosylation of flavonoid and isoflavone glycosides is one of the most extensively investigated transformation processes in LAB-mediated plant-food fermentation. As shown in Table 2, representative microbial transformation reactions and quantitative parameters involved in plant bioactive compound bioconversion are summarized.

Soybean isoflavone transformation represents a typical example. *Lactobacillus paraplantarum* KM exhibited strong β-glucosidase-associated isoflavone-converting activity during soymilk fermentation, achieving 100% hydrolysis of genistin and approximately 90% hydrolysis of daidzin within 6 h. Consequently, the concentrations of the corresponding aglycones, daidzein and genistein, increased approximately 6- and 7-fold, respectively, compared with their initial levels [53]. Similarly, LAB fermentation increased daidzein and genistein proportions from 14.24% and 6.89% to 36.20% and 28.80% of total isoflavones after 24 h fermentation [54]. More recent studies have further demonstrated that *Lactiplantibacillus plantarum* strains can efficiently promote the bioconversion of isoflavone glucosides into their corresponding aglycones during soymilk fermentation, highlighting the importance of strain-specific β-glucosidase activity in isoflavone transformation [55].

In addition to flavonoid conversion, LAB modify phenolic acids through decarboxylation and reduction reactions. Phenolic acid decarboxylases from *Lactiplantibacillus plantarum* and *Lactobacillus brevis* convert hydroxycinnamic acids into vinyl derivatives. Under optimized conditions, *L. plantarum* converted ferulic acid into 4-vinylguaiacol [56,57].

LAB fermentation can also enhance phenolic compound release through organic acid production and matrix disruption. Plant-based fermentation with LAB has been reported to increase extractable phenolic contents depending on substrate characteristics and fermentation conditions [58]. Therefore, LAB-mediated transformation provides a mild and food-compatible strategy for improving the accessibility and functional potential of plant bioactive.

### 3.2. Bacillus-Mediated Transformation Strategies

*Bacillus* species represent important microbial platforms for plant bioactive compound transformation due to their strong extracellular enzyme secretion capacity and ability to degrade complex plant matrices. Food-associated strains, including *Bacillus subtilis*, *B. licheniformis*, and *B. amyloliquefaciens*, have been widely applied in soybean and cereal fermentations [22,59]. Their transformation ability is mainly attributed to extracellular enzymes, including proteases, cellulases, xylanases, tannases, and glycosidases, which facilitate plant matrix degradation and phytochemical release.

A major advantage of *Bacillus*-mediated fermentation is its capacity to transform bound phenolic compounds associated with dietary fibers and cell wall components. Tannase-producing *Bacillus* strains hydrolyze tannins into smaller phenolic molecules, such as gallic acid, through ester bond cleavage, thereby increasing the availability of phenolic compounds [25]. Similarly, β-glucosidase-producing strains can catalyze the hydrolysis of flavonoid and isoflavone glycosides, promoting the formation of corresponding aglycones during plant substrate fermentation.

Beyond phytochemical transformation, *Bacillus* fermentation integrates protein conversion with bioactive peptide production. Solid-state fermentation of soybean meal using *B. subtilis* SBM_1_ increased peptide yield to 220.93 mg/g after 12 h fermentation, with peptides below 3000 Da accounting for 71.64% of total products, while protease activity reached 103.37–241.93 U/g under optimized conditions (45 °C, 25% inoculum, material/water ratio 1:1.25) [60]. These results indicate that *Bacillus* fermentation can simultaneously enhance phytochemical accessibility and generate functional protein-derived ingredients.

In addition, *Bacillus* fermentation may produce functional metabolites, including γ-aminobutyric acid (GABA), antimicrobial peptides, and organic acids. However, industrial application requires strict strain selection because safety characteristics vary among species. Therefore, only food-grade strains with confirmed genomic safety and absence of virulence-associated genes should be considered for functional food production.

### 3.3. Yeast-Mediated Transformation of Plant Bioactive Compounds

Yeasts are important microbial platforms for transforming plant-derived bioactive compounds due to their metabolic flexibility, environmental adaptability, and diverse enzymatic systems. Food-associated yeasts, particularly *Saccharomyces cerevisiae*, possess various hydrolytic and oxidative enzymes, including β-glucosidases, esterases, and oxidoreductases, which contribute to the structural modification of phenolics and other phytochemicals. Unlike simple degradation, yeast-mediated biotransformation enables selective conversion of conjugated compounds into more bioavailable derivatives.

β-Glucosidase-mediated deglycosylation is one of the most extensively investigated yeast transformation pathways. During soybean fermentation, β-glucosidase-producing yeasts can efficiently hydrolyze isoflavone glucosides, such as daidzin and genistin, resulting in increased levels of corresponding aglycones, including daidzein and genistein. In fermented soymilk, LAB–yeast co-fermentation achieved high conversion efficiencies of daidzin and genistin, reaching 62.7–92.3% and 97.5–98.5%, respectively, after 24–48 h fermentation. These findings demonstrate the potential of LAB–yeast co-fermentation as an effective strategy for enhancing the bioavailability and functional properties of soybean isoflavones [61].

Engineered yeast systems have further improved transformation efficiency. *Saccharomyces cerevisiae* displaying β-glucosidase from *Aspergillus oryzae* efficiently hydrolyzed soybean isoflavone glycosides, reducing residual glycosides to approximately 6.2% and enabling enhanced production of isoflavone aglycones [62]. In addition, non-*Saccharomyces* yeasts exhibit diverse enzymatic activities, including esterase and oxidoreductase functions, which contribute to phenolic modification and aroma-related metabolite formation. However, their industrial application still requires optimization of conversion yield, fermentation scale, and strain safety evaluation. Overall, yeast-mediated transformation provides a mild and effective strategy for improving the accessibility and functional properties of plant bioactive compounds, with demonstrated conversion efficiencies from laboratory to potential food-processing applications.

### 3.4. Filamentous Fungi-Mediated Transformation of Plant Bioactive Compounds

Filamentous fungi represent efficient microbial platforms for plant bioactive compound transformation due to their extensive extracellular enzyme secretion, broad substrate adaptability, and diverse oxidative metabolism. Species belonging to *Aspergillus*, *Monascus*, and *Rhizopus* have been widely applied in food fermentation and biotechnology [63,64]. Compared with bacteria and yeasts, filamentous fungi possess complex enzyme systems, including glycosidases, hydrolases, and cytochrome P450 monooxygenases, enabling diverse modifications such as deglycosylation, hydroxylation, and oxidation–reduction reactions [65]. Therefore, fungal fermentation has been investigated as an effective approach for transforming structurally complex plant metabolites.

#### 3.4.1. Aspergillus-Mediated Transformation

Food-grade *Aspergillus oryzae* is one of the most extensively studied fungi for phytochemical transformation. Its extracellular β-glucosidases, cellulases, and esterases promote plant matrix degradation and conversion of conjugated phytochemicals into more accessible derivatives.

Quantitative studies have demonstrated the efficiency of *A. oryzae*-mediated isoflavone transformation. Fermentation of soybean germ flour with *A. oryzae* KACC 40247 produced 165 mg/g isoflavone aglycones under optimized conditions (7% substrate concentration, pH 6.0, 33 °C, 24 h fermentation), with a productivity of approximately 479 mg/L/h [66]. These results indicate that *A. oryzae* fermentation can effectively enhance phytochemical accessibility through enzymatic deglycosylation.

#### 3.4.2. Monascus-Mediated Transformation and Functional Metabolite Production

The genus *Monascus* has attracted considerable attention as a food-associated fungal platform because of its ability to transform plant substrates and produce functional metabolites, including pigments and monacolin K [67,68]. During solid-state fermentation, *Monascus* secretes carbohydrases, proteases, and glycosidases that contribute to plant matrix degradation, phenolic release, and phytochemical remodeling [69].

Quantitative studies have demonstrated the technological potential of *Monascus* fermentation. In soybean-based fermentation, *M. purpureus* significantly increased isoflavone aglycone formation and antioxidant activity after approximately 15 days of fermentation, indicating enhanced conversion of conjugated phytochemicals into more accessible derivatives [70].

During fermentation, β-glucosidase-mediated deglycosylation and hydrolytic enzyme-mediated matrix degradation contribute to phytochemical remodeling. In addition, the secondary metabolic pathways of *Monascus* can be influenced by plant-derived substrates, which regulate pigment biosynthesis and metabolite profiles [71,72,73,74].

#### 3.4.3. Rhizopus and Other Filamentous Fungi

Species belonging to the genus Rhizopus are widely used in traditional fermented foods, particularly tempeh, due to their strong proteolytic activity and carbohydrate-degrading capacity. During fermentation, *Rhizopus oligosporus* produces abundant extracellular proteases that hydrolyze plant proteins into peptides and free amino acids, contributing to the formation of bioactive peptide fractions with potential antioxidant and functional properties [75]. In addition to proteases, enzymes produced by *R. oligosporus*, including cellulases, xylanases, and β-glucosidases, participate in plant matrix degradation and glycosidic bond hydrolysis, thereby facilitating the release of phenolic compounds and isoflavone aglycones [76]. Soybean fermentation by *R. oligosporus* has been reported to increase phenolic accessibility through enhanced conversion of conjugated phytochemicals into smaller molecular forms [77].

In addition to *Rhizopus*, other filamentous fungi, including *Penicillium*, *Mucor*, and related genera, exhibit considerable transformation capabilities toward diverse plant secondary metabolites. Their cytochrome P450 monooxygenase systems enable regioselective oxidation and hydroxylation reactions, allowing structural modifications of flavonoids, terpenoids, and alkaloids that are difficult to achieve using conventional chemical approaches [78].

Overall, filamentous fungi represent versatile microbial platforms for the transformation of plant-derived bioactive compounds due to their extensive extracellular enzyme secretion, complex oxidative metabolism, and diverse catalytic pathways. However, their application requires strict safety evaluation. Food-associated species, such as *Aspergillus oryzae* and selected *Penicillium* strains, should be preferentially employed because of their established history of safe use in food fermentation. Potential risks associated with mycotoxin-producing species should be controlled through genome-based screening, analysis of toxin biosynthetic gene clusters, and metabolomic profiling. Similarly, *Bacillus* strains intended for functional food applications should be carefully evaluated to exclude virulence-associated genes, toxigenic potential, and transferable antibiotic resistance determinants. Therefore, integrating catalytic performance with comprehensive safety assessment during microbial strain selection is essential for the industrial development of microbial-transformed food ingredients.

### 3.5. Molecular Mechanisms of Microbial Transformation of Plant Bioactive Compounds

These mechanisms mainly include deglycosylation, ester hydrolysis, oxidation–reduction, methylation, and glycosylation. As shown in Figure 2, these enzyme-mediated reactions can alter the structures of plant-derived bioactive compounds and further affect their physicochemical properties, bioavailability, and biological activities.

#### 3.5.1. Deglycosylation and Glycosidic Bond Hydrolysis

Deglycosylation is one of the most extensively investigated microbial transformation pathways involved in the modification of plant bioactive compounds [58,79]. During fermentation, microbial β-glucosidases hydrolyze C–O glycosidic bonds, converting glycosylated compounds into their corresponding aglycones and free sugars. Representative transformations include the conversion of rutin to quercetin, daidzin to daidzein, and ginsenoside Rb1 to compound K through sequential glycosidic bond cleavage [80,81]. These reactions have been reported in β-glucosidase-producing microorganisms, including lactic acid bacteria, *Saccharomyces cerevisiae*, *Metschnikowia pulcherrima*, *Aspergillus oryzae*, and *Rhizopus oligosporus*. The extent of deglycosylation is influenced by strain-dependent enzyme activity, substrate structure, and fermentation conditions.

#### 3.5.2. Esterification and Hydrolysis Reactions

Esterification and hydrolysis reactions regulate the release and modification of ester-bound plant metabolites. Many phenolic compounds in plant tissues are linked to organic acids, sugars, or cell wall polymers through ester bonds, limiting their extraction and accessibility.

Microbial esterases and tannases catalyze ester bond cleavage, releasing free phenolic acids and related derivatives. For example, esterase-mediated hydrolysis of chlorogenic acid produces caffeic acid and quinic acid. Similarly, tannase-producing microorganisms can convert hydrolyzable tannins into smaller phenolic compounds, such as gallic acid [82]. These reactions have been demonstrated in food-associated microorganisms, including *Bacillus* species, lactic acid bacteria, and filamentous fungi, in which extracellular esterases facilitate the transformation of bound phenolics during fermentation.

#### 3.5.3. Oxidation and Reduction Reactions

Microbial oxidation–reduction reactions generate structural diversity by modifying hydroxyl groups, aromatic rings, and side-chain structures of plant metabolites. These transformations are mainly catalyzed by oxidoreductases, including laccases, peroxidases, alcohol dehydrogenases, and cytochrome P450 monooxygenases [83,84]. Representative reactions include hydroxylation of aromatic compounds, reduction of carbonyl groups, and oxidative modification of phenolic structures [65]. Compared with hydrolysis reactions, oxidation–reduction pathways provide broader structural modification capacity and are particularly important in filamentous fungi and engineered microbial systems [85].

#### 3.5.4. Methylation and Glycosylation

Microbial methylation and glycosylation represent important enzymatic strategies for increasing structural diversity and optimizing the functional characteristics of plant bioactive compounds [86,87]. Microbial methyltransferases transfer methyl groups from donor molecules, such as S-adenosylmethionine (SAM), to hydroxyl or amino groups of phytochemicals, producing methylated derivatives with altered chemical properties [88]. Glycosyltransferases catalyze the transfer of sugar residues to specific positions of acceptor molecules, generating glycosylated derivatives with modified polarity and stability [89].

## 4. Enhanced Bioactivities of Microbial-Transformed Plant Compounds

Microbial transformation can significantly influence the biological performance of plant-derived compounds by generating metabolites with altered molecular properties and functional characteristics. Although many phytochemicals exhibit promising activities in vitro, their application in functional foods is often restricted by limitations related to stability, absorption, and metabolic behavior. Through microbial-mediated structural modification, plant compounds can be converted into derivatives with improved accessibility and biological potential.

The functional enhancement of microbial-transformed compounds has been mainly associated with improved antioxidant activity, regulation of inflammatory responses, metabolic modulation, neuroprotective effects, and interactions with gut microbiota.

### 4.1. Enhancement of Antioxidant Activity Through Microbial Transformation

Oxidative stress caused by excessive accumulation of reactive oxygen species (ROS) is closely associated with cellular damage and is considered an important factor influencing aging processes and various chronic conditions. Plant-derived polyphenols are widely recognized as natural antioxidants due to their hydroxyl groups, conjugated structures, and electron-donating abilities. However, their practical antioxidant performance in biological systems is often restricted by poor absorption, rapid metabolism, and limited stability.

Microbial transformation can enhance the antioxidant capacity of plant-derived compounds by generating structurally modified metabolites with increased accessibility of functional groups and improved biological availability [42]. In addition to hydrolysis reactions, microbial oxidation, hydroxylation, and methylation can generate modified phenolic derivatives with altered redox properties [22]. These transformations have been experimentally demonstrated in selected microbial systems, although their contribution to antioxidant enhancement requires further validation using cellular and in vivo models. Overall, microbial transformation represents an effective approach for producing plant-derived ingredients with improved antioxidant performance, while future studies should focus on establishing quantitative relationships between specific structural modifications and antioxidant outcomes [33].

### 4.2. Improvement of Bioavailability and Intestinal Absorption

Limited bioavailability remains one of the major challenges restricting the application of many plant-derived bioactive compounds in functional foods. Numerous phytochemicals exhibit poor water solubility, extensive intestinal metabolism, and limited membrane permeability, which reduce their absorption and utilization efficiency.

Microbial transformation can improve the bioaccessibility of plant compounds by generating metabolites with altered molecular characteristics and potentially enhanced intestinal availability [90,91]. Microbial transformation of ginsenosides represents a well-established example of improving the absorption potential of plant-derived compounds. Selective hydrolysis of glycosidic moieties by microbial enzymes generates minor ginsenosides, including compound K, which generally exhibit higher intestinal absorption and biological activity than their parent compounds [92].

However, conversion into aglycones or deglycosylated derivatives does not necessarily indicate an inevitable improvement in bioavailability. Actual bioavailability is influenced by multiple factors, including aqueous solubility, intestinal metabolism, transporter activity, gut microbiota interactions, and host metabolic processes [93]. Therefore, future studies should integrate chemical structural characterization with evaluations of in vitro digestion, intestinal bioaccessibility, cellular uptake, and pharmacokinetic investigations in animal and clinical models.

### 4.3. Enhancement of Anti-Inflammatory Activity

Chronic low-grade inflammation is closely associated with metabolic imbalance and impaired physiological regulation. Many plant-derived compounds, particularly polyphenols and flavonoids, possess anti-inflammatory potential; however, their effectiveness may be restricted by limited bioavailability and insufficient cellular accessibility. Microbial transformation can enhance the functional properties of these compounds by generating derivatives with improved molecular interactions and biological accessibility.

Microbial-transformed metabolites may regulate inflammation-related pathways, including NF-κB, MAPK, and Nrf2/ARE signaling, thereby modulating the production of inflammatory mediators such as TNF-α, IL-6, IL-1β, and NO [92,94]. Structural modifications generated through microbial transformation, including deglycosylation, oxidation, and reduction, may alter the bioactivity of plant-derived metabolites and contribute to enhanced anti-inflammatory potential. However, most current evidence is derived from in vitro cellular models, and further in vivo studies are required to clarify the contribution of specific transformation pathways to anti-inflammatory effects and establish causal relationships between metabolite structures and biological outcomes [95].

### 4.4. Regulation of Glucose and Lipid Metabolism

Metabolic disorders, including impaired glucose homeostasis, insulin resistance, and abnormal lipid accumulation, represent important targets for functional food development. However, the application of many plant-derived bioactive compounds is restricted by limited bioavailability and insufficient biological accessibility.

Microbial transformation enhances the metabolic regulatory potential of phytochemicals by generating more accessible phenolic acids, flavonoid aglycones, and other low-molecular-weight metabolites [96]. These transformed compounds may improve the inhibition of carbohydrate-hydrolyzing enzymes, particularly α-amylase and α-glucosidase, thereby contributing to the regulation of postprandial glucose release [97].

In addition, microbial-transformed metabolites may influence lipid metabolism through AMPK activation, PPAR signaling regulation, and modulation of adipocyte differentiation. Fermented soybean products provide a representative example, in which microbial conversion increases isoflavone aglycones and bioactive peptides, contributing to improved glucose and lipid metabolic regulation [98].

### 4.5. Modulation of Gut Microbiota by Microbial-Transformed Compounds

The interaction between dietary bioactive compounds and gut microbiota has emerged as an important mechanism underlying the functional properties of plant-based foods. Microbial transformation establishes a dynamic relationship between microorganisms, plant compounds, and intestinal microbial ecosystems. On one hand, food-associated microorganisms convert structurally complex phytochemicals into smaller and more bioaccessible metabolites; on the other hand, these transformed compounds can further influence gut microbial composition and metabolic activity.

Microbial-transformed phenolic metabolites can also serve as substrates for beneficial intestinal microorganisms, including *Lactobacillus*, *Bifidobacterium*, and *Akkermansia*, promoting microbial balance and intestinal homeostasis [99]. Transformation-derived metabolites, such as phenolic acids and short-chain fatty acids (SCFAs), may contribute to epithelial barrier maintenance, immune regulation, and microbial community stability. Studies have shown that fermentation-derived polyphenol metabolites can modify gut microbial diversity and increase the abundance of SCFA-producing bacteria [51]. Therefore, microbial transformation may enhance the functionality of plant-based foods through both direct improvement of phytochemical properties and indirect regulation of gut microbiota-mediated responses.

### 4.6. Enhancement of Neuroprotective and Anti-Aging Effects

Oxidative stress, mitochondrial dysfunction, and chronic inflammation are important factors associated with neuronal aging and age-related functional decline. Plant-derived flavonoids and phenolic compounds exhibit neuroprotective potential; however, their effectiveness is often limited by poor bioavailability and rapid metabolism.

Microbial transformation can generate structurally modified flavonoids and phenolic metabolites with improved accessibility and altered biological activity. These transformed compounds may enhance cellular defense by regulating antioxidant and stress-response pathways, particularly the Nrf2/ARE signaling pathway, thereby reducing oxidative damage and inflammatory responses [100].

In addition, microbial-derived metabolites may influence longevity-associated pathways, including AMP-activated protein kinase (AMPK) and silent information regulator 1 (SIRT1) signaling, which are involved in mitochondrial function, cellular stress resistance, autophagy, and metabolic regulation [101]. Although these effects have been mainly demonstrated in cellular and animal models, further in vivo and clinical studies are required to clarify the neuroprotective and anti-aging potential of microbial-transformed plant compounds.

## 5. Applications of Microbial-Transformed Plant Compounds in Functional Food Development

The increasing demand for natural and health-oriented foods has promoted the development of functional foods containing plant-derived bioactive compounds. Microbial transformation provides an effective approach to improve the technological and functional properties of plant compounds by generating structurally modified derivatives through enzymatic conversion. Therefore, microbial-transformed plant ingredients have become promising components for fermented foods, nutraceutical products, and next-generation functional food formulations.

### 5.1. Fermented Plant-Based Beverages Enriched with Microbial-Transformed Bioactive Compounds

Plant-based fermented beverages, including fruit-, vegetable-, tea-, and botanical-based products, represent important applications of microbial transformation in functional food development [102]. These substrates contain abundant phenolics, flavonoids, and anthocyanins; however, many compounds occur in conjugated or polymeric forms that limit their bioaccessibility. Microbial fermentation can convert these compounds into more accessible derivatives through enzymatic reactions such as glycoside hydrolysis, ester cleavage, and oxidation, thereby improving the functional characteristics and application value of fermented beverages [58].

LAB- and yeast-mediated fermentation of berry, grape, and citrus substrates has been reported to increase free phenolic compounds and modify flavonoid profiles through microbial enzymatic transformation [58]. These findings demonstrate that microbial fermentation can be applied to develop beverage products enriched with transformed phytochemicals and diversified metabolite profiles.

Tea-based fermented beverages provide another representative example of microbial-transformed functional foods. Products such as kombucha and dark tea involve complex microbial communities composed of bacteria and yeasts, which transform tea polyphenols through oxidation, hydrolysis, and other metabolic reactions. During kombucha fermentation, the symbiotic culture of bacteria and yeasts (SCOBY) converts tea-derived compounds into organic acids and diverse phenolic metabolites, resulting in altered chemical profiles and improved functional properties [103]. These processes highlight the potential of microbial fermentation for developing beverage products with modified phytochemical compositions and enhanced technological value.

### 5.2. Fermented Cereals and Legume-Based Functional Foods

Cereals and legumes are important substrates for microbial transformation because they contain abundant proteins, polysaccharides, dietary fibers, and phytochemicals. However, many phenolic compounds and flavonoids are present in bound forms associated with cell wall structures, limiting their accessibility. Microbial fermentation provides an effective approach for modifying these food matrices by releasing bound compounds and generating structurally transformed metabolites through enzymatic reactions [104].

Cereal fermentation represents another important application of microbial transformation. In whole grains, many phenolic acids are covalently bound to cell wall polysaccharides. Microbial enzymes, including feruloyl esterases, xylanases, and cellulases, facilitate the release of bound phenolic compounds through ester bond cleavage and polysaccharide degradation. Sourdough fermentation by LAB and yeasts has been reported to increase free phenolic acids, including ferulic acid and caffeic acid, through microbial enzymatic activity. In addition, fermentation can reduce antinutritional components such as phytic acid through microbial phytase activity, supporting the development of cereal- and legume-based functional foods with improved nutritional characteristics [105].

### 5.3. Fermented Plant Extracts and Nutraceutical Ingredients

Beyond traditional fermented foods, microbial transformation has increasingly been applied to the production of high-value plant-derived nutraceutical ingredients. Compared with conventional extraction approaches, microbial conversion enables selective modification of plant metabolites and the production of structurally transformed compounds with improved technological properties and application potential.

*Lactobacillus pentosus* DC101 isolated from kimchi has been reported to efficiently convert ginsenoside Rd into compound K through sequential glycosidic hydrolysis [106]. This bioconversion pathway involves the intermediate formation of ginsenoside F2, highlighting the potential of food-associated microorganisms for producing high-value minor ginsenosides.

Microbial transformation has also been applied to flavonoid-rich plant extracts. Citrus flavonoids, such as hesperidin and naringin, can be converted into their corresponding aglycones, hesperetin and naringenin, through microbial α-L-rhamnosidase- and β-glucosidase-mediated hydrolysis reactions. In addition, plant ingredients containing soy isoflavones, tea polyphenols, curcuminoids, and anthocyanins have been investigated as potential substrates for microbial conversion, providing opportunities for developing functional ingredients with enhanced accessibility and diversified metabolite profiles [107]. These approaches expand the range of transformed plant-derived ingredients available for functional food and nutraceutical applications.

Overall, microbial transformation provides a versatile platform for producing customized plant-derived nutraceutical ingredients. Integration of strain selection, enzyme discovery, and metabolic engineering approaches may further improve the precision and scalability of microbial conversion processes for functional food applications.

### 5.4. Bee-Derived Functional Foods Enhanced by Microbial Transformation

Bee-derived products, including bee pollen, royal jelly, honey, and propolis, contain diverse nutrients and bioactive components, such as proteins, amino acids, polysaccharides, and phenolic compounds. However, the application of these products in functional foods can be limited by the low accessibility of some bioactive constituents due to complex food matrices. Microbial transformation has therefore been investigated as an approach to modify bee-derived substrates through targeted enzymatic reactions and microbial metabolism, thereby generating more accessible metabolites and value-added ingredients [108].

Among bee-derived products, bee pollen is considered a suitable substrate for microbial transformation because of its abundant proteins, carbohydrates, lipids, vitamins, minerals, and phytochemicals. Natural fermentation during bee bread formation involves a diverse microbial community composed mainly of lactic acid bacteria and yeasts, which contributes to pollen preservation, degradation of pollen components, and release of embedded nutrients and bioactive compounds [109,110]. Inspired by this natural process, controlled fermentation using selected microorganisms, including LAB, *Bacillus* species, and yeasts, has been explored as an approach to improve the nutritional accessibility and functional properties of bee pollen [111].

Recent studies have shown that specific microbial strains can modify bee pollen components through enzyme-mediated reactions. For example, solid-state fermentation using a microbial consortium containing *Lactiplantibacillus plantarum*, *Apilactobacillus kunkeei*, and *Lactobacillus acidophilus* promoted the transformation of pollen phenolic compounds. These transformations were accompanied by increased levels of polyphenols, flavonoids, ellagic acid, kaempferol, quercetin, and quercetin-3-O-rutinoside, although the magnitude of these changes varied depending on fermentation conditions and microbial composition [112]. Similarly, mixed fermentation with yeasts and LAB increased total phenolic content and altered metabolite profiles compared with unfermented bee pollen [113]. These effects were mainly associated with microbial enzyme activities, including β-glucosidase-, protease-, and esterase-mediated conversion processes [114].

In addition to bee pollen, fermentation of royal jelly and other bee-derived products has been explored for the development of value-added functional foods. Microbial proteases can modify protein components into smaller peptides, while esterases and other metabolic enzymes may contribute to the transformation of lipid-associated compounds. Furthermore, co-fermentation of bee-derived products with plant substrates provides a potential strategy for integrating microbial transformation of plant phytochemicals with the nutritional characteristics of bee products.

### 5.5. Development of Probiotic and Potential Synbiotic Functional Foods

Microbial-transformed plant compounds provide new opportunities for developing probiotic and potential synbiotic functional foods by combining plant-derived substrates with beneficial microorganisms. Fermented foods containing viable probiotic microorganisms and microbial-derived metabolites represent complex functional systems in which microbial metabolism contributes to phytochemical transformation and product diversification [115]. According to the ISAPP scientific definition, synbiotic products require the combination of probiotics with selectively utilized substrates that confer a demonstrated health benefit. Therefore, not all fermented foods containing plant compounds and microorganisms can be classified as synbiotic products. Plant-based substrates fermented with LAB and other probiotic microorganisms may serve as promising platforms for developing synbiotic foods when the plant-derived components are demonstrated to selectively support beneficial microbial activity [102]. During fermentation, probiotic microorganisms can transform complex phytochemicals through enzymatic reactions, generating metabolites such as phenolic acids, flavonoid derivatives, and other low-molecular-weight compounds. These microbial-transformed metabolites may further interact with intestinal microorganisms, providing potential mechanisms linking fermented foods with gut microbiota-mediated functions [116].

The combination of probiotics and transformed plant metabolites provides a promising strategy for developing multifunctional fermented foods. Fermentation-derived metabolites, including phenolic acids and short-chain fatty acids (SCFAs), may contribute to interactions between dietary components and intestinal microbial ecosystems. Therefore, microbial transformation serves as a technological link connecting plant substrates, probiotic microorganisms, and microbiota-oriented functional food development.

### 5.6. Industrial Challenges and Future Perspectives

Although microbial transformation has been successfully demonstrated in various laboratory-scale functional food applications, industrial implementation remains limited by process variability, insufficient standardization, and difficulties in maintaining consistent metabolite production. Transformation efficiency depends on microbial strain characteristics, substrate composition, enzyme activity, and fermentation parameters, which collectively influence metabolite profiles and product reproducibility [117]. Therefore, standardized fermentation protocols, optimized strain combinations, and controlled bioprocessing strategies are essential for achieving reliable transformation outcomes.

Safety evaluation represents another critical consideration for microbial-transformed functional foods. Although many food-associated microorganisms have established histories of safe use, newly generated metabolites require comprehensive assessment, including chemical characterization, biological activity evaluation, toxicity testing, and regulatory compliance before commercialization [118]. In addition, potential interactions between microbial-derived metabolites and host physiology require further investigation.

Scale-up from laboratory fermentation to industrial production remains a major technological challenge. Differences in oxygen transfer, mass transport, substrate heterogeneity, and process control between laboratory and industrial systems may significantly affect microbial activity, conversion efficiency, and product quality. Future industrial development requires improved bioreactor design, real-time monitoring technologies, and quantitative optimization of fermentation parameters to ensure stable and reproducible production.

Future advances in microbial-transformed functional foods will depend on improved strain selection, enzyme discovery, metabolic engineering, and multi-omics-guided process optimization. These approaches may enable more precise regulation of microbial conversion pathways and targeted production of specific metabolites [119,120]. However, most engineered microbial platforms and precision biomanufacturing strategies remain at developmental stages and require further validation regarding safety, regulatory acceptance, and large-scale food applications.

## 6. Multi-Omics, Synthetic Biology, and Future Perspectives of Microbial Transformation of Plant Bioactive Compounds

### 6.1. Multi-Omics-Based Elucidation of Microbial Transformation Mechanisms

Although microbial transformation has been widely applied in fermented foods, the molecular mechanisms underlying plant bioactive compound conversion remain incompletely understood. Traditional studies mainly focused on changes in total metabolites or functional activities, whereas the relationships among microbial strains, catalytic enzymes, metabolic pathways, and transformation products require further clarification [121].

Recent advances in multi-omics technologies, including genomics, transcriptomics, proteomics, metabolomics, and microbiomics, provide powerful approaches for systematically characterizing microbial transformation processes. Advances in microbial genome sequencing have facilitated the discovery of plant bioactive compound-transforming enzymes. Genome mining has been experimentally validated for identifying functional genes encoding β-glucosidases, esterases, tannases, phenolic acid decarboxylases, cytochrome P450 monooxygenases, glycosyltransferases, and methyltransferases involved in deglycosylation, hydrolysis, oxidation, and glycosylation reactions. Like, genomic analyses of lactic acid bacteria have identified β-glucosidase genes involved in flavonoid glycoside conversion, while fungal genome mining has revealed diverse P450 systems for modifying flavonoids and terpenoids. These findings provide valuable resources for enzyme discovery and strain selection. However, engineered microbial platforms and synthetic biocatalytic systems remain emerging strategies requiring further validation for large-scale applications [122]. Transcriptomic and proteomic analyses further reveal microbial responses to plant substrates and environmental conditions, providing insights into the regulation of transformation-associated pathways. Metabolomics based on LC–MS/MS, GC–MS, and NMR platforms facilitates the identification of intermediate and final transformation products, allowing reconstruction of metabolic pathways and discovery of bioactive metabolites [123].

Integration of multi-omics datasets provides a comprehensive framework for linking microbial genetic potential with enzyme activity and metabolite production. However, many current studies remain descriptive, and further validation of enzyme–substrate relationships and metabolic networks is required to establish precise transformation mechanisms.

### 6.2. Genome-Based Safety Evaluation of Functional Food Microorganisms

Synthetic biology provides new strategies for improving microbial transformation efficiency through enzyme engineering, metabolic pathway modification, and genome editing. Engineering of transformation-related enzymes can enhance catalytic activity, substrate specificity, and stability, whereas metabolic engineering enables redirection of microbial flux toward desired products. CRISPR-based genome editing further facilitates precise regulation of genes involved in plant compound conversion [120].

In addition to single-strain engineering, synthetic microbial consortia have emerged as promising systems for complex substrate transformation. Compared with conventional fermentation using individual strains, artificial microbial communities can combine complementary metabolic functions through microbial division of labor. For example, yeasts may contribute to plant matrix degradation, whereas lactic acid bacteria and other microorganisms participate in subsequent phenolic compound conversion. Nevertheless, industrial application of engineered strains and synthetic consortia remains limited by challenges associated with stability, reproducibility, safety, and regulatory acceptance.

### 6.3. Transcriptomics Reveals Regulatory Mechanisms During Biotransformation

The increasing availability of multi-omics and fermentation datasets has promoted the application of artificial intelligence (AI) and machine learning approaches in microbial transformation. These technologies can integrate genomic information, metabolomic profiles, and fermentation parameters to predict microbial performance, identify functional enzymes, and optimize transformation conditions [124]. However, most AI-assisted strategies remain at the prediction stage, and experimental validation is still required before practical industrial implementation.

Despite significant progress, large-scale application of microbial transformation remains challenging. Variations in microbial strains, substrate composition, reactor conditions, oxygen transfer, and fermentation parameters can influence transformation efficiency and product consistency. Therefore, standardized fermentation protocols, quantitative process control, and economic evaluation are essential for improving reproducibility, scalability, and commercial feasibility.

### 6.4. Metabolomics for Tracking Transformation Pathways and Bioactive Metabolites

The successful application of microbial-transformed plant compounds in functional foods requires comprehensive safety evaluation of both production microorganisms and newly generated metabolites. Genome-based assessment enables the identification of undesirable genetic traits, including antibiotic resistance genes, virulence-associated factors, and toxin-related pathways, thereby supporting the selection of suitable food-grade microorganisms [125].

In addition, transformation-derived metabolites require independent evaluation regarding chemical identity, stability, toxicity, allergenicity, and biological effects. Although many microbial-transformed compounds exhibit promising bioactivities in vitro, further animal studies and clinical investigations are needed to confirm their physiological relevance and support health claims. Regulatory frameworks, such as GRAS (Generally Recognized as Safe) and QPS (Qualified Presumption of Safety) systems, provide guidance for microbial safety assessment; however, evaluation criteria for newly generated transformation products require further development [126].

### 6.5. Microbiomics and Synthetic Microbial Consortia

Future research on microbial transformation should focus on establishing more predictable and controllable conversion systems through the integration of multi-omics, synthetic biology, and computational approaches. Precision microbial transformation based on strain selection, enzyme characterization, and pathway optimization may enable targeted production of valuable compounds, including flavonoid aglycones, modified phenolics, and rare saponin derivatives [127].

Artificial microbial communities represent another promising direction for improving transformation of complex plant substrates, although optimization of community stability and reproducibility remains necessary. Furthermore, AI-driven digital fermentation may facilitate real-time prediction and optimization of microbial processes by integrating multidimensional biological and process data [128].

Personalized functional foods represent an emerging application area, where microbial transformation may generate metabolite profiles tailored to individual physiological responses. Future studies integrating plant resources, microbial biotechnology, and functional food development may expand the application potential of microbial-transformed bioactive compounds.

## 7. Conclusions and Outlook

Microbial transformation represents a sustainable and promising biotechnological strategy for enhancing the application value of plant-derived bioactive compounds. Through selective enzymatic modification and metabolic remodeling, microorganisms can convert structurally complex phytochemicals into more accessible derivatives with improved physicochemical properties and potentially enhanced biological functions. Food-associated microorganisms, including lactic acid bacteria, *Bacillus* species, yeasts, and filamentous fungi, possess diverse catalytic capabilities that enable various transformation reactions, such as deglycosylation, hydrolysis, oxidation–reduction, methylation, and glycosylation. These microbial-mediated processes have been successfully applied to the structural modification of flavonoids, isoflavones, phenolic acids, terpenoids, saponins, and other plant secondary metabolites, providing new opportunities for the development of functional foods, nutraceuticals, and high-value bioactive ingredients.

Despite considerable advances, the broader industrial application of microbial transformation remains limited by several critical challenges, including incomplete understanding of transformation mechanisms, insufficient process standardization, safety evaluation of newly generated metabolites, and difficulties associated with large-scale production. Variations in microbial strains, substrate composition, fermentation conditions, and analytical approaches have hindered direct comparison among different transformation systems. Therefore, establishing standardized evaluation frameworks based on conversion efficiency, product yield, productivity, and process stability is essential. In parallel, the relationship between structural modification and biological functionality requires further clarification through integrated approaches combining chemical characterization, bioaccessibility assessment, cellular studies, animal models, and clinical investigations.

Future development of microbial transformation will increasingly depend on the integration of multi-omics technologies, synthetic biology, metabolic engineering, and artificial intelligence. Precision microbial transformation strategies involving rational strain selection, enzyme engineering, and pathway optimization may enable targeted and efficient production of valuable derivatives, including flavonoid aglycones, modified phenolic compounds, and rare saponin metabolites. In addition, artificial microbial consortia and AI-assisted digital fermentation platforms provide new opportunities for improving the transformation of complex plant substrates and optimizing bioprocess performance. However, further studies are required to validate the stability, reproducibility, safety, and industrial feasibility of these emerging technologies.

Emerging applications, including personalized functional foods and the utilization of novel plant-derived resources, represent promising future directions. Microbial transformation may facilitate the generation of fermented products with specific metabolite profiles tailored to individual physiological responses. Nevertheless, the complex interactions among transformed metabolites, gut microbiota, and host health outcomes require further investigation through well-designed clinical studies. Furthermore, plant resources such as urban melliferous plants and bee-derived products, including bee pollen, contain diverse phytochemicals and represent potential substrates for microbial transformation. Integrating plant resource characterization, microbial biotechnology, and functional food engineering may provide new strategies for developing sustainable and high-value food ingredients.

Overall, microbial transformation serves as an important technological bridge connecting plant resources, microbial metabolism, and functional food innovation. Advances in microbial ecology, enzyme discovery, computational biology, and fermentation engineering will facilitate the establishment of safer, more efficient, and standardized transformation platforms. Through interdisciplinary integration, microbial-transformed plant bioactive compounds are expected to play an increasingly important role in the development of next-generation sustainable functional foods and nutraceutical products.

## Figures and Tables

**Figure 1:**
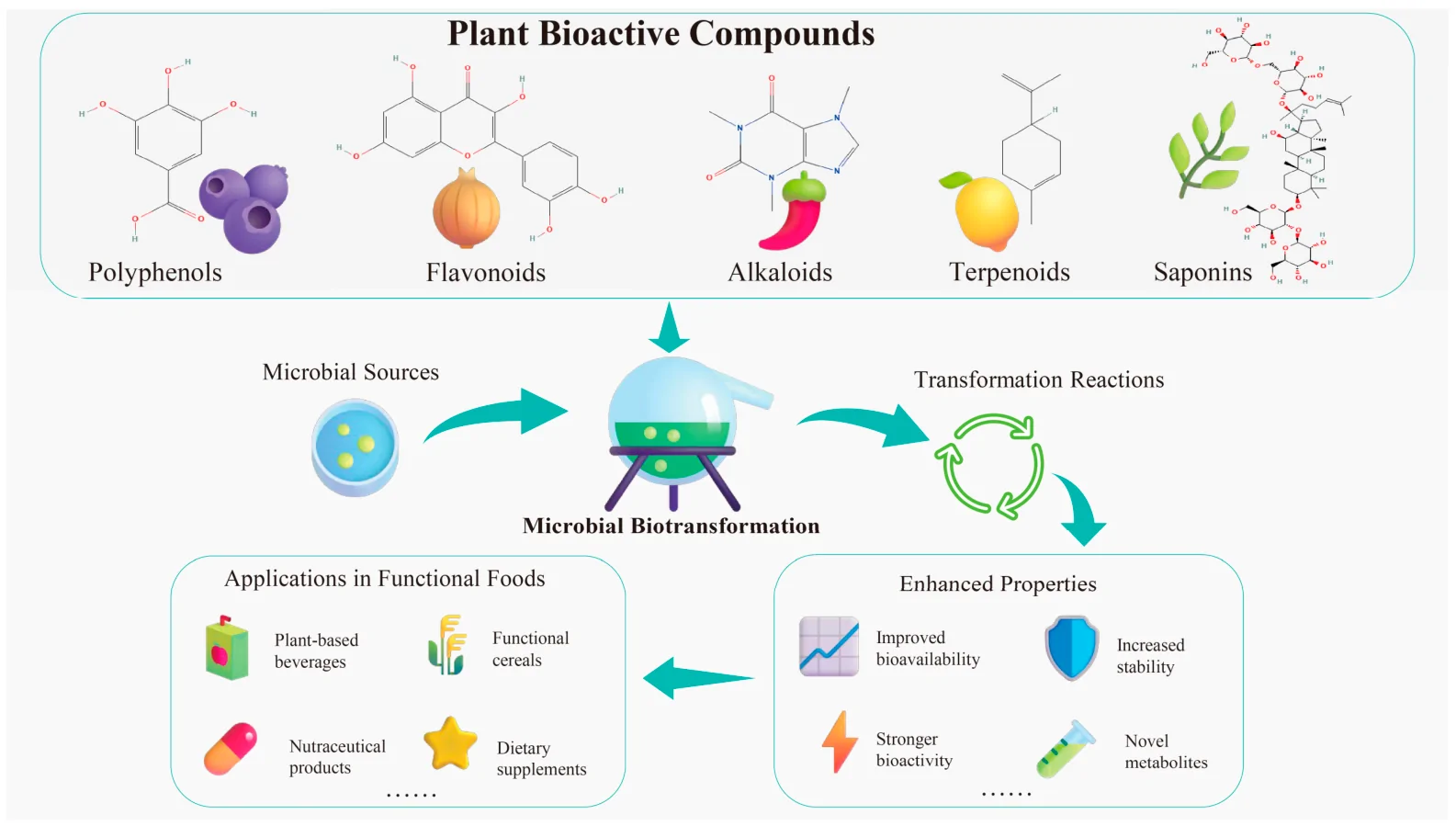
Overview of microbial biotransformation of plant bioactive compounds for functional food development.

**Figure 2:**
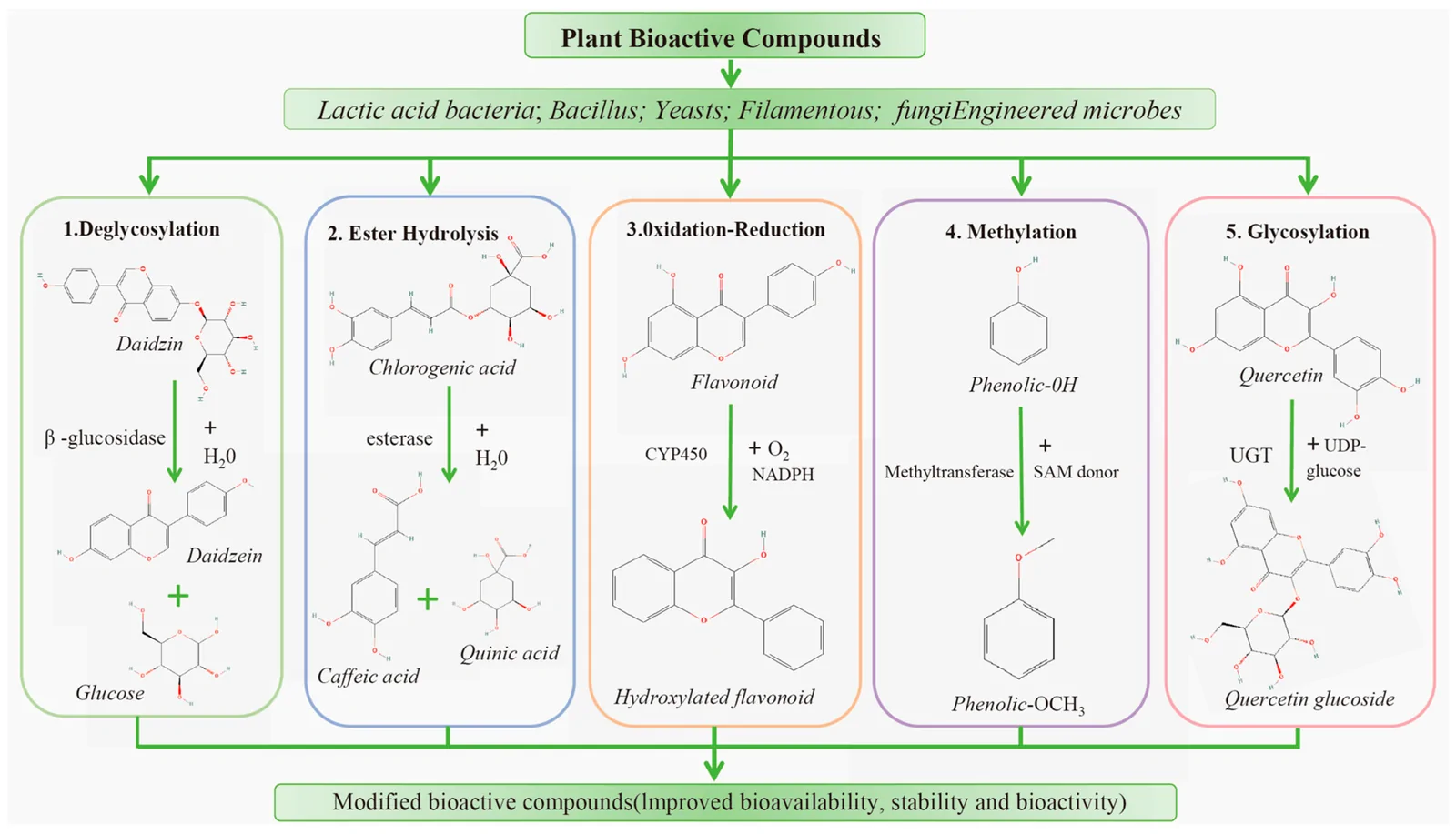
Representative pathways of plant bioactive compound transformation by microbial enzymes.

**Table 2:** Representative microbial transformation reactions and quantitative parameters involved in plant bioactive compound bioconversion.

Microbial Group	Representative Transformation Reaction (Chemical Equation)	Key Enzymes	Substrate → Product	Conversion Efficiency/Productivity	Fermentation Conditions
*Lactic acid bacteria* (LAB)	Daidzin + H_2_O → Daidzein + Glucose	β-Glucosidase; phenolic acid decarboxylase	Soybean isoflavone glycosides → isoflavone aglycones; hydroxycinnamic acids → vinyl derivatives	*L. paraplantarum* KM achieved 100% hydrolysis of genistin and approximately 90% hydrolysis of daidzin within 6 h; daidzein and genistein increased approximately 6- and 7-fold, respectively	Approximately 6 h for *L. paraplantarum* KM; 24 h fermentation for other LAB-mediated isoflavone transformation
*Bacillus* species	Protein + H_2_O → Peptides + Amino acids Genistin + H_2_O → Genistein + Glucose	Proteases; tannases; β-glucosidases	Soybean proteins → bioactive peptides; bound phenolics/isoflavone conjugates → released bioactive derivatives	*B. subtilis* SBM_1_ fermentation produced 220.93 mg/g peptides, with peptides below 3000 Da accounting for 71.64% of total products; protease activity reached 103.37–241.93 U/g	Solid-state fermentation; 45 °C; 12 h; 25% inoculum; material/water ratio 1:1.25
*Yeasts*	Genistin + H_2_O → Genistein + Glucose	β-Glucosidase; esterases; oxidoreductases	Isoflavone glycosides → isoflavone aglycones	In LAB–yeast co-fermentation, daidzin conversion reached 62.7–92.3% and genistin conversion reached 97.5–98.5% after 24–48 h	Approximately 28–30 °C; 24–48 h fermentation
*Filamentous fungi* *(Aspergillus)*	Genistin + H_2_O → Genistein + Glucose	β-Glucosidase; cellulase; esterase	Bound soybean isoflavones → free isoflavone aglycones	*A. oryzae* KACC 40247 fermentation produced 165 mg/g isoflavone aglycones with a productivity of approximately 479 mg/L/h	33 °C; 24 h; substrate concentration 7%; pH 6.0
*Monascus* spp. *(M. purpureus)*	Isoflavone glycosides + H_2_O → Isoflavone aglycones + sugars	β-Glucosidases; glycosidases; proteases; carbohydrases	Soybean isoflavone glycosides → free isoflavone aglycones; plant phytochemicals → modified/released phenolic compounds and functional metabolites	Enhanced isoflavone aglycone formation and antioxidant activity during soybean fermentation	Solid-state fermentation; approximately 15 days
*Rhizopus* spp.	Soybean protein + H_2_O → Peptides + Amino acids; Isoflavone glycosides + H_2_O → Aglycones + sugars	Proteases; β-glucosidases; cellulases; xylanases	Soybean proteins → peptides and free amino acids; conjugated phytochemicals → smaller phenolic derivatives and isoflavone aglycones	Increased accessibility of phenolic compounds and formation of bioactive peptide fractions	Fermentation conditions are substrate- and strain-dependent

## Data Availability

No new data were created or analyzed in this study. Data sharing is not applicable to this article.
